# Admissions for chronic ambulatory care sensitive conditions - a useful measure of potentially preventable admission?

**DOI:** 10.1186/s12913-015-1137-0

**Published:** 2015-10-16

**Authors:** Jo M. Longman, Megan E. Passey, Dan P. Ewald, Elizabeth Rix, Geoffrey G. Morgan

**Affiliations:** University Centre for Rural Health – North Coast, University of Sydney, PO Box 3074, Lismore, NSW 2480 Australia; North Coast Primary Health Network, Lismore, 2480 Australia

**Keywords:** Avoidable admission, Preventable admission, Potentially preventable hospitalisation, Patient admission (statistics & numerical data), Ambulatory care sensitive conditions

## Abstract

**Background:**

Potentially preventable hospital admission (an admission deemed to be potentially preventable given appropriate care in the community-based healthcare setting) has been a topic of international research attention for almost three decades. Recently this has been largely driven by the imperative to reduce ever-increasing unplanned hospital admissions. However, identifying potentially preventable admissions is difficult. As a result, the population level indicator of admissions for ambulatory care sensitive conditions (ACSCs) has been used as a proxy measure for potentially preventable admission. The adoption of this measure has become common, and in Australia, the rate of admissions for chronic ACSCs is now an important component of measuring health system performance and accountability, and is directly linked to funding. Admission for a chronic ACSC is also used to identify individuals for targeting of interventions to reduce preventable admissions.

**Discussion:**

Hospital admission for chronic ACSCs is a population measure based on admission diagnoses, it therefore should not be used to identify individual preventable admissions. At present we are unable to determine individual admissions that are deemed to be preventable or, therefore, articulate the factors associated with admissions which are preventable.

**Summary:**

As we are currently unable to identify individual admissions that are preventable, little is understood about the underlying causes and factors contributing to preventable admissions. A means of assessing preventability of individual admissions is required. Only then can we explore the antecedents, and patient and clinician perspectives on preventable admissions. Until we have a clearer understanding of this, our capacity to inform policy and program development remains compromised.

## Background

Potentially preventable admission (an admission deemed to be potentially preventable given appropriate care in the community-based healthcare setting) has been a topic of considerable international research attention for almost three decades. More recently, the imperative to reduce ever-increasing unplanned hospital admissions has been the main driver of this research, along with the assumption that a significant proportion of these potentially preventable admissions, particularly for chronic conditions, might be prevented with intervention prior to the admission. Reducing the number of admissions might greatly benefit patients and their families, as well as the health care system, although it is important to note that reductions in admissions do not necessarily reflect improved clinical outcomes.

### Ambulatory care sensitive conditions

Potentially preventable admissions are difficult to define and measure. In research, policy development and program design, one well-used proxy measure for potentially preventable admissions is admissions for “ambulatory care sensitive conditions” (ACSC), a concept first introduced in New York in the early 1990s as an indicator of population level access to outpatient care [1]. More recently it has been suggested that rates of ACSC reflect quality of community-based care, rather than access, particularly in settings with universal health care [2]. ACSCs fall into three categories: vaccine preventable, acute conditions, and chronic conditions. Although variously defined, generally they are those conditions which respond well to interventions deliverable in community-based healthcare settings, and if managed well should not require hospital admission. Chronic ACSCs such as congestive heart failure and chronic obstructive pulmonary disease (COPD) make up the largest proportion of all ACSC admissions in Australia, particularly amongst older people [3]. In Australia the rate of ACSCs is an important component of measuring health system performance and accountability [4], and variation in rates of ACSC admissions are assumed to reflect variation in access to, or quality of, community-based care. While this assumption has been challenged in both Canada [2, 5] and Australia [6] ACSC continues to be used as a performance indicator and funding measure and is also being used in program implementation to identify individuals for targeting of interventions to reduce admissions [7].

Our aim in this Debates paper is to highlight the need for a better measure than the population level indicator of ACSC to identify potentially preventable admissions for individuals.

## Discussion

### Difficulties with using admissions for ambulatory care sensitive conditions to identify potentially preventable admissions

The number of admissions for chronic ACSCs is a population level measure based on specific admission diagnoses and therefore cannot assess the preventability of individual admissions. Policy development and program design has failed to take account of this in using ACSC as a mechanism for identifying preventable admissions and targeting of health interventions.

One of the major difficulties with using ACSC admission as a proxy for preventable admission is that not all admissions for chronic ACSCs are potentially preventable. For example, even with management of heart failure following evidence-informed guidelines, patients’ condition will gradually deteriorate and may eventually require admission. Therefore using chronic ACSC admissions overestimates the rate of preventable admission by capturing an unknown number of admissions that are necessary and could not feasibly have been prevented.

There are several implications of being unable to assess the preventability of individual admissions. Firstly, the factors associated with admissions which are deemed to be preventable cannot be identified. Secondly, it remains unclear whether any chronic ACSC admissions are more preventable than others. Thirdly it is not possible to explore how the preventability of individual admissions varies across different population groups or in different contexts. Put together, this means we have limited evidence for developing and targeting interventions to reduce preventable admissions. Furthermore, timescale is complex in assessing the preventability of a chronic ACSC admission. For example a chronic ACSC admission of an older patient admitted for an exacerbation of their COPD may have been preventable with appropriate care/intervention decades previously in supporting their smoking cessation, or rather in the weeks or days prior to the admission with appropriate clinical treatment of the exacerbation.

### Gaps in current knowledge

#### ACSC admission and community-based care

There has been little detailed work on the use of community-based healthcare services in the weeks or days leading up to an admission, or the patients’ and their care-givers’ decision-making processes and actions that contributed to that admission [8]. Some evidence exists showing that community-based healthcare interventions targeting patients with specific chronic ACSCs can result in reductions in admissions particularly for COPD and asthma [9–12]. This suggests that some admissions may indeed be preventable with improved community-based services, although few studies have used hospital admission as an outcome measure and generally the evidence for interventions in community-based healthcare settings is too limited to draw any conclusions [13]. In addition, evaluations of community-based interventions to reduce ACSC admissions which use a before and after model following individual patients, generally fail to account for the natural regression to the mean of hospitalisation rates, i.e. hospital admissions of individuals generally reduce to a lower frequency after a peak period even without interventions [14, 15].

#### Understanding complexity

It is widely recognised that overall health and health related behaviours are determined by complex socio-economic, cultural, individual and health service delivery factors including variations in admission policy between hospitals and in admission thresholds amongst clinicians [16]. However, few studies on potentially preventable admission have attempted to understand this complexity.

#### Assessing the preventability of individual admissions

In recent years our research team has explored hospital admission amongst older people with chronic ACSCs in rural Australia [17–19]. One third of the Australian population lives outside major cities [20] and the rate of ACSC admissions is particularly burdensome in these areas [3, 21–23]. Our work has highlighted the need to quantify and describe individual admissions that are preventable. This will provide the basis for improved understanding of the antecedents to a preventable admission and how they may differ from a chronic ACSC admission which is not preventable. Understanding the factors contributing to preventable admissions at the individual level is essential in order to more efficiently target interventions aimed at reducing these admissions.

We have been unable to identify any validated tools to assess the preventability of individual admissions. There have been several clinical decision-making support tools developed to assess the clinical appropriateness of admissions on the day of admission [24–27], however, the difference between ‘appropriate’ admission (requires admission on the day) and ‘preventable’ (not managed well in the community leading up to the admission) is marked [28]. A recent systematic review of studies exploring readmissions deemed preventable, highlighted the paucity of robust research in this area with regard to defining preventability. It reported considerable variability between studies in the proportion of individual admissions deemed preventable, and concluded that the proportion of readmissions which are potentially preventable remains unknown [29].

### Future directions

Our group has piloted the use of a tool to assess the preventability of individual admissions, based on the assessment of the senior nurse caring for the patient and the physician under whom the patient was admitted. The tool, based on an extensive literature review and consultation with clinicians, draws on the earlier work of Oddone et al. [28] and Arozullah et al. [30] but can be used by clinicians at the time of admission, rather than using a retrospective audit process. It also considers individual and social factors more extensively than this previous work and defines the timeframe for preventability as the previous three months. We have determined that a three month time frame prior to admission is reasonable for this purpose, based on consultation with clinicians and other researchers, and the need to identify a period in which it is reasonable to expect any interventions aimed at reducing preventable admissions to be effective.

Face and content validity of the tool has been tested using a panel of expert clinicians. Minor amendments to the content and item order of the tool followed this validation process. The feasibility and acceptability of using the tool was assessed in three sites, and demonstrated its utility. The tool aims to capture some of the complexity around the confluence of circumstances that brings individuals to the point of admission, and not rely entirely on the use of diagnostic categories or clinical notes. Our future work aims to conduct a more detailed validation of the tool and to further understand the patient, carer and community-based healthcare providers’ (including family physicians’) perspectives on admissions.

In the meantime, the rate of admissions for chronic ACSCs is used in the measurement of health system performance directly linked to funding, and interventions have been designed and implemented to reduce potentially preventable admissions, on the basis of limited understanding. For example, in Australia sizeable state-wide programs have been implemented which target patients with chronic ACSC admissions and involve a broad variety of interventions including a focus on improving coordination of health services. This assumes that the underlying problem is lack of coordination of services. The evidence to support this assumption is limited, with two recent large trials finding no impact of care coordination on the use of acute care [31, 32].

## Conclusion 

In summary, in the absence of a measure to assess the preventability of individual unplanned admissions for chronic conditions, little is understood about the complex underlying causes and factors contributing to individual admissions deemed preventable, hampering efforts to develop and target interventions to reduce these admissions. A means of assessing the preventability of individual admissions, is required. Only then can we explore the antecedents, and patient, caregiver and clinician perspectives of admissions deemed preventable. Until we have a clearer understanding of this, we are in a compromised position to inform policy and program development and to measure the right outcomes in this important area.
